# Supplementary material for: Identification of potential dual -targets anti- toxoplasma gondii compounds through structure-based virtual screening and in-vitro studies
Source: PLoS One. 2020 May 22;15(5):e0225232. doi: 10.1371/journal.pone.0225232 (PMC7244133; doi:10.1371/journal.pone.0225232)
Supplement: S1 Checklist — (DOC) [file pone.0225232.s001.doc]

**The ARRIVE Checklist**

**TITLE**

**1 Provide as accurate and concise a description of the content of the article as possible.**

Identification of Potential Dual-Targets Anti- *Toxoplasma gondii* Compounds Through Structure-Based Virtual Screening and In-Vitro Studies

**ABSTRACT**

**2 Provide an accurate summary of the background, research objectives (including details of the species or strain of animal used), key methods, principal findings, and conclusions of the study.**

*Toxoplasma gondii* is the etiologic agent of toxoplasmosis, a disease which can lead to morbidity and mortality of the fetus and immunocompromised individuals. Due to the limited effectiveness or side effects of existing drugs, the search for better drug candidates is still ongoing. In this study, we performed structure-based screening of potential dual-targets inhibitors of active sites of *T. gondii* drug targets such as uracil phosphoribosyltransferase (UPRTase) and adenosine kinase (AK). First screening of virtual compounds from the National Cancer Institute (NCI) was performed *via* molecular docking. Subsequently, the hit compounds were tested *in-vitro* for anti- *T. gondii* effect using cell viability assay with Vero cells as host to determine cytotoxicity effects and drug selectivities. Clindamycin, as positive control, showed a selectivity index (SI) of 10.9, thus compounds with SI > 10.9 specifically target *T. gondii* proliferation with no significant effect on the host cells. Good anti- *T. gondii*  effects were observed with NSC77468 (7-ethoxy-4-methyl-6,7-dihydro-5H -thiopyrano[2,3- d ] pyrimidin-2-amine) which showed SI values of 25. This study showed that *in-silico* selection can serve as an effective way to discover potentially potent and selective compounds against *T. gondii.*

**INTRODUCTION**

**Background**

**3 a. Include sufficient scientific background (including relevant references to previous work) to understand the motivation and context for the study, and explain the experimental approach and rationale.**

**b. Explain how and why the animal species and model being used can address the scientific objectives and, where appropriate, the study’s relevance to human biology.**

The use of the animal in an earlier part of the study is solely to maintain the tachyzoites by passaging using intraperitoneal inoculation into healthy Swiss albino mice. Once the tachyzoites harvested from the mice, the tachyzoites were used in the in-vitro study. The obtained tachyzoites from the experimental model mimic a natural physiologic host-parasite interaction (Sher, Tosh, & Jankovic, 2017). Therefore, it will ensure the pathogenicity of the tachyzoites when used in the in-vitro study to test for the anti-*T. gondii* effect using cell culture assay.

**Objectives**

**4 Clearly describe the primary and any secondary objectives of the study, or specific hypotheses being tested.**

In this study, virtual screening of anti- *T. gondii* was used to screen for potential drug candidates that have good complementarity in terms of shape and physico-chemical properties with the selected drug targets. The National Cancer Institute (NCI), USA database was used to virtually screen for anti- *T. gondii*. Subsequently pure compounds of the *in-silico* hits were then obtained from the NCI for *in vitro* validation. The selected pure compounds from the NCI database were tested using cell viability assay with Vero cells as host to determine cytotoxicity effects and drug selectivity.

**METHODS**

**Ethical statement**

**5 Indicate the nature of the ethical review permissions, relevant licenses (e.g. Animal [Scientific Procedures] Act 1986), and national or institutional guidelines for the care and use of animals, that cover the research.**

The study was carried out according to Universiti Sains Malaysia (USM) ethical guidelines (USM/PPSF50 (003) JLD2) under internationally accepted principles for laboratory animal use and care approved by USM Institutional Animal Care and Use Committee.

**Study design**

**6 For each experiment, give brief details of the study design, including:**

**a. The number of experimental and control groups.**

**b. Any steps taken to minimise the effects of subjective bias when allocating animals to treatment (e.g., randomisation procedure) and when assessing results (e.g., if done, describe who was blinded and when).**

**c. The experimental unit (e.g. a single animal, group, or cage of animals).**

**A time-line diagram or flow chart can be useful to illustrate how complex study designs were carried out.**

a. For each experiment, a group of four female Swiss mice was used for maintenance of *T. gondii* RH strain. There was no control group involve since the purpose of the experimental was only to maintain tachyzoites through intraperitoneal passages in mice.

b – c. Not applicable

**Experimental procedures**

**7 For each experiment and each experimental group, including controls, provide precise details of all procedures carried out. For example:**

**a. How (e.g., drug formulation and dose, site and route of administration, anaesthesia and analgesia used [including monitoring], surgical procedure, method of euthanasia). Provide details of any specialist equipment used, including supplier(s).**

**b. When (e.g., time of day).**

**c. Where (e.g., home cage, laboratory, water maze).**

**d. Why (e.g., rationale for choice of specific anaesthetic, route of administration, drug dose used).**

Intraperitoneal injection – (Inoculation of 3ml of (3 x 10^5^ /ml) of RH tachyzoites)

- Mice is removed from the cage and restrain appropriately in the head‐down position.
- Area to be injected (lower right quadrant of the abdomen) is sterilized with 70% ethanol.
- A 27G needle will be inserted into the identified area, and the plunger will be pulled back slightly to ensure negative pressure. If there is no negative pressure, the plunger will be then depressed to inject the desired amount of antigen.

All mice were sacrificed at four days post-inoculation by cervical dislocation followed by harvesting of tachyzoites from the peritoneal cavity.

**Experimental animals**

**8 a. Provide details of the animals used, including species, strain, sex, developmental stage (e.g., mean or median age plus age range), and weight (e.g., mean or median weight plus weight range).**

**b. Provide further relevant information such as the source of animals, international strain nomenclature, genetic modification status (e.g. knock-out or transgenic), genotype, health/immune status, drug- or test naıve, previous procedures, etc.**

Female Swiss albino mice (6-8 weeks old) were purchased from Animal Research and Service Centre (ARASC), Universiti Sains Malaysia.

**Housing and husbandry**

**9 Provide details of:**

**a. Housing (e.g., type of facility, e.g., specific pathogen free (SPF); type of cage or housing; bedding material; number of cage companions; tank shape and material etc. for fish).**

**b. Husbandry conditions (e.g., breeding programme, light/dark cycle, temperature, quality of water etc. for fish, type of food, access to food and water, environmental enrichment).**

**c. Welfare-related assessments and interventions that were carried out before, during, or after the experiment.**

1. Housing: type of cage- open polycarbonate cages; bedding material-wood shaving; number of cage companion 5 cages
2. Husbandry condition: no specific husbandry or environmental enrichment was necessary; food: Standardized mouse diet (Altromin)
3. Not applicable

**Sample size**

**10 a. Specify the total number of animals used in each experiment and the number of animals in each experimental group.**

**b. Explain how the number of animals was decided. Provide details of any sample size calculation used.**

**c. Indicate the number of independent replications of each experiment, if relevant.**

A total of 12 mice were used in the study (4 per each experimental group) to obtained enough tachyzoites from the mice.

**Allocating animals to experimental groups**

**11 a. Give full details of how animals were allocated to experimental groups, including randomisation or matching if done.**

**b. Describe the order in which the animals in the different experimental groups were treated and assessed.**

A group of four mice were injected with tachyzoites for each inoculation cycles. The process was performed continuously until all the 12 mice were used (3 cycles).

**Experimental outcomes**

**12 Clearly define the primary and secondary experimental outcomes assessed (e.g., cell death, molecular markers, behavioural changes).**

The percent cell viability was proportional to the mortality of *T. gondii.* SI (selectivity index) measures the strength of inhibition against the Vero cells and the *T. gondii.* High SI indicates that the compound specifically targets the proliferation of *T. gondii* with negligible effect on Vero cells

**Statistical methods**

**13 a. Provide details of the statistical methods used for each analysis.**

**b. Specify the unit of analysis for each dataset (e.g. single animal, group of animals, single neuron).**

**c. Describe any methods used to assess whether the data met the assumptions of the statistical approach.**

No statistical analysis was used in these animal studies.

**RESULTS**

**Baseline data
14 For each experimental group, report relevant characteristics and health status of animals (e.g., weight, microbiological status, and drug- or test-naıve) before treatment or testing (this information can often be tabulated).**

All animals analyzed were in good health.

**Numbers analysed**

**15 a. Report the number of animals in each group included in each analysis. Report absolute numbers (e.g. 10/20, not 50%).**

**b. If any animals or data were not included in the analysis, explain why.**

Not applicable.

**Outcomes and estimation**

**16 Report the results for each analysis carried out, with a measure of precision (e.g., standard error or confidence interval).**

Not applicable.

**Adverse events**

**17 a. Give details of all important adverse events in each experimental group.**

**b. Describe any modifications to the experimental protocols made to reduce adverse events.**

There were no adverse events.

**DISCUSSION**

**Interpretation/scientific implications**

**18 a. Interpret the results, taking into account the study objectives and hypotheses, current theory, and other relevant studies in the literature.**

Not applicable.

**b. Comment on the study limitations including any potential sources of bias, any limitations of the animal model, and the imprecision associated with the results.**

Not applicable.

**c. Describe any implications of your experimental methods or findings for the replacement, refinement, or reduction (the 3Rs) of the use of animals in research.**

The virulent *T. gondii* tachyzoites cannot be obtained freshly for *in-vitro* study.

**Generalisability/translation**

**19 Comment on whether, and how, the findings of this study are likely to translate to other species or systems, including any relevance to human biology.**

Not applicable.

**Funding**

**20 List all funding sources (including grant number) and the role of the funder(s) in the study.**

This study was partially funded by Science Fund from the Ministry of Science and Innovation, No. 02-01-05-SF0428, and Universiti Sains Malaysia 1001/PFARMASI/870031. NHS was supported by the Malaysian Institute of Pharmaceuticals and Nutraceuticals through Agilent Bio-analytical Industrial Training Program (BIDP).

Reference:

Sher, A., Tosh, K., & Jankovic, D. (2017). Innate recognition of Toxoplasma gondii in humans involves a mechanism distinct from that utilized by rodents*. Cell Mol Immunol,* 14(1), 36-42.
